# Low prevalence of human leukocyte antigen-B*5701 in HIV-1-infected Chinese subjects: a prospective epidemiological investigation

**DOI:** 10.1186/s12981-015-0064-9

**Published:** 2015-08-19

**Authors:** Hongwei Zhang, Tong Zhang, Hongxin Zhao, Ning Han, Haiwei Zhou, Yun He, Qingxia Zhao, Hong Li, Huiqin Li, Mi Zhang, Jianjian Li, Yongtao Sun, Ke Zhao, Qing Liu, Zhiying Liu, Zhen Li, Wei Xia, Yun Lan, Haolan Hu, Weiping Cai, Hao Wu

**Affiliations:** Center for Infectious Diseases, Beijing You’an Hospital, Capital Medical University, 8 Xitoutiao, Youanmenwai St., Fengtai Dist., Beijing, 100069 People’s Republic of China; Department of Infectious Diseases, Beijing Ditan Hospital, Capital Medical University, Beijing, 100069 China; Department of Infectious Diseases, The 6th People Hospital of Zhengzhou, Zhengzhou, 450015 China; Yunnan AIDS Care Center, Kunming, 650301 China; Department of Infectious Diseases, Tangdu Hospital, The Fourth Military Medical University, Xi’an, 710038 China; Department of Infectious Diseases, Guangzhou Eighth People’s Hospital, Guangzhou, 510060 China

**Keywords:** HLA-B*5701, HIV, Prevalence

## Abstract

**Background:**

Human leukocyte antigen (HLA)-B*5701 is strongly associated with developing a hypersensitivity reaction to abacavir (ABC). Limited data exist on HLA-B*5701 prevalence in HIV-1-infected subjects in China. We investigated HLA-B*5701 prevalence in HIV-1-infected population including Han and non-Han ethnic groups.

**Methods:**

A prospective multi-centre study was designed to determine status of HLA-B*5701 in HIV-1-infected adults at six sites across China. HLA-B*5701 was tested by the method of PCR-SSP.

**Results:**

From six centers, 3,000 HIV-infected patients [2,452 (81.7%) Han, 548 (18.3%) Non-Han] were recruited with a mean age of 36.7 years old. The overall HLA-B*5701 prevalence was 0.86% [95% confidence interval (CI) 0.55–1.26%]. The prevalence of HLA-B*5701 among Han subjects was similar to that among non-Han subjects, which was 0.88% (95% CI 0.54–1.34%) and 0.76% (95% CI 0.19–1.93%), respectively (*p* value = 0.787). There were no differences in prevalence of HLA-B*5701 between subjects born in Henan, Yunnan, Shanxi, Guangdong, Hebei, Beijing and other provinces (*p* = 0.999).

**Conclusions:**

HLA-B*5701 prevalence is very low in HIV-infected Chinese subjects, both in the Han and Non-Han nationalities. And there are no differences among different birthplaces across China.

## Background

The major histocompatibility complex allele human leukocyte antigen (HLA)-B*5701 has been strongly associated with the risk of hypersensitivity reaction to abacavir (ABC). A prospective, randomized, multicenter, double-blind study showed that prospective HLA-B*5701 screening can reduce the incidence of hypersensitivity reaction to ABC [1]. In addition, the presence of HLA-B*5701 was associated with clinically diagnosed hypersensitivity reaction in Hispanic and Thai patients infected with HIV [2]. Since prospectively excluding HLA-B*5701-positive patients from receiving ABC can eliminate immunologically confirmed hypersensitivity reaction and significantly reduce the rate of diagnosis of clinical hypersensitivity reaction, recent guidelines strongly recommend screening for HLA-B*5701 before starting patients on an ABC-containing regimen [3, 4].

Carriage rates of HLA-B*5701 vary greatly across the globe. The Caucasian population in the United Kingdom, the USA and Australia shows a prevalence of 5–8%. In contrast, the HLA-B*5701 is found in less than 1% of the black population in sub-Saharan Africa [5]. Previous studies reported that East Asians, including Taiwanese, Japanese, Chinese, and Koreans, had a low prevalence of HLA-B*5701 [6–8]. The allele frequency of HLA-B*5701 in Chinese patients was reported to be less than 1% in various ethnic groups [9]. However, the patients were relatively small in number and they limited to some local areas. Before ABC is widely used in China, it is necessary to further explore the prevalence of HLA-B*5701 in China on a large scale.

## Methods

### Study design

This was a prospective multicenter epidemiological study in HIV infected patients. From December 2012 to March 2013, 3,000 HIV-1 infected patients were recruited from 6 centers located in 5 cities of China (Beijing, Zhengzhou, Kunming, Guangzhou and Xi’an). Of the six treatment centers, Zhengzhou Sixth Hospital, Guangzhou Eighth Hospital and Yunnan AIDS Care Center were the largest infectious hospitals in provinces with highest HIV/AIDS prevalence. Xi’an Tangdu Hospital provided care to HIV/AIDS patients from western area of China. Beijing You’an Hospital and Beijing Ditan Hospital were two of the largest infectious diseases hospitals, caring over 80% of HIV infected patients in Beijing. They all had experiences in national HIV/AIDS care and treatment programs. As a leading center, Beijing You’an Hospital also had experiences in international clinical trials, such as TMC 278-C204, -C215 and -C222.

HIV-1-infected patients over the age of 18 years were eligible for the study, including both treatment naïve and treatment-experienced patients. The study was approved by the Beijing You’an Hospital Ethics Committee. Written consent was obtained prior to any study related activities.

After signing the informed consent, subjects were interviewed to collect demographic data, including age, gender, ethnicity, HIV transmission route and treatment history. CD4 cell counts and HIV viral loads within last 3 months were also collected if available. Blood samples were collected during the visit for HLA-B* 5701 genotyping.

### HLA-B* 5701 typing

DNA was extracted from blood samples using the QIAmp DNA Mini Kit (Qiagen, Canada). HLA-B* 5701 genotyping was determined by the method of PCR-SSP using the commercial kit of BF-10-11(Abbott Laboratories, America). According to the instruction manual, 10 μL sterile distilled water added to the lyophilized PCR reaction solution containing the allele-specific primers, internal control primer and the PCR reaction Taq polymerase needed, dNTPs, buffer, MgCl_2_, and the dye loading buffer (loading buffer). Then, 10 μL DNA sample with concentration of 5–10 ng/μL was added to each well, followed by the procedures PCR reaction. The 5–10 μL PCR products were electrophoresed on a 2% agarose gel. Positive samples were confirmed using the same method. The detection of HLA-B* 5701 typing was monitored by a third party, Beijing Anapure Bioscientific technology co., LTD.

### Statistical analysis

There was a descriptive analysis of the parameters about the demographic characteristics of the subjects. Frequency of a positive HLA-B*5701 status was detected among Han and non-Han populations and in different regions of China. As the prevalence of HLA-B*5701 was very low, the confidence interval (CI) was calculated using the Wilson score [10]. The Fisher’s exact test was used to compare HLA-B*5701 prevalence between populations or birthplaces. All data were analyzed with SPSS version 17.0 (SPSS, Chicago, IL, USA).

## Results

HLA-B*5701 status was determined among 3,000 subjects who provided informed consent for study participation. At the time of enrolment, the mean age of study participants was 36.7 years [standard deviation (SD) 10.2 years], with the majority being male (77.9%). Approximately, 81.7% of subjects were Han nationalities, while non-Han nationalities only accounted for 18.3%. There were 2,889 patients who had information of CD4 cell counts, with the mean (±SD) value of 367.8 ± 201.5 cells/mm^3^, and 2,130 patients who had information of viral load, with the mean (±SD) value of 2.30 ± 1.23 log copies/mL.

In this study, the overall prevalence of HL-AB*5701 was 0.86% (95% CI 0.55–1.26%). The prevalence of HLA-B*5701 for Han nationality was 0.88% (95% CI 0.54–1.34%) and for non-Han nationalities it was 0.76% (95% CI 0.19–1.93%) (Table 1). The non-Han ethnic group included Dai, Bai, Jingpo, Man, Hui, Mongol, Korean, Zhuang, Daur, Gejia, Lisu, Buyi, Miao, Dong, and Russian. Since the number of other minorities was too small, the prevalence of HLA-B*5701 in each ethnic group was not analyzed.

**Table 1:** Prevalence of human leukocyte antigen (HLA)-B*5701 of all subjects by main ethnic groups

	N	HLA-B*5701 status			Positive HLA-B*5701 status		*P* value
		Positive	Negative	Undetermined	%	95% exact CI	
Han	2,452	21	2,373	58	0.88	0.54–1.34	0.787
Non-Han	548	4	524	20	0.76	0.19–1.93	
Overall	3,000	25	2,897	78	0.86	0.55–1.26	

N = number of enrolled subjects; % = positive cases/number of subjects with available results × 100; 95% CI = 95% lower and upper confidence limits.

The most frequent reported birthplace was Henan (20.3%), followed with Yunnan (16.1%), Shanxi (12.2%), Guangdong (11.8%), Hebei (9.9%), Beijing (6.33%) and other provinces (23.37%). For subjects born in these provinces, the prevalence of HLA-B*5701 was 0.84% (95% CI 0.28–1.96%), 0.85% (95% CI 0.23–2.15%), 0.85% (95% CI 0.18–2.45%), 0.87% (95% CI 0.18–2.52%), 0.69% (95% CI 0.08–2.47%), 1.08% (95% CI 0.13–3.81%) and 0.88% (95% CI 0.32–1.90%), respectively. There were no differences in prevalence of HLA-B*5701 between subjects born in different places (P = 0.999) (Table 2).

**Table 2:** Prevalence of human leukocyte antigen (HLA)-B*5701 by birthplace

Birthplace	N	HLA-B*5701 status			Positive HLA-B*5701 status		*P* value
		Positive	Negative	Undetermined	%	95% exact C I (%)	
Henan	610	5	588	17	0.84	0.28–1.96	0.999
Yunnan	483	4	467	12	0.85	0.23–2.15	
Shanxi	365	3	352	10	0.85	0.18–2.45	
Guangdong	355	3	341	11	0.87	0.18–2.52	
Hebei	296	2	288	6	0.69	0.08–2.47	
Beijing	190	2	184	4	1.08	0.13–3.81	
Other provinces	701	6	677	18	0.88	0.32–1.90	

N = number of enrolled subjects; % = positive cases/number of subjects with available results × 100; 95% CI = 95% lower and upper confidence limits.

The amplification bands were very weak in some patients, even though we followed the protocol strictly and repeated. According to the company’s instruction, we defined them as “undetermined results” showing in Tables 1 and 2.

## Discussion

The present study showed the prevalence of HLA-B*5701 (0.86%) was much lower in HIV-infected patients in China mainland when compared with that (5–8%) in Western countries [11–13]. This is similar to the result (0.48%) from a previous study in 1,043 cases of HIV-infected injection drug users in Sichuan province [14]. A previous study in Taiwan showed that there was only one individual expressing HLA-B*5701 in 320 HIV infected patients [6]. The prevalence of HLA-B*5701 in Chinese patients of other areas was even lower. It was 0.17% in Hong Kong Chinese patients (572 patients) and 0% in Singapore Chinese patients (149 patients) [15].

Similar result was also found in non-Han Chinese populations. China is composed of 56 ethnic groups. Han Chinese account for 91.51% of the overall Chinese population and the other 55 make up the remaining 8.49% according to the Sixth National Population Census of 2010 [16]. Of the 528 non-Han ethnic patients with HIV infection for whom the HLA-B*5701 test was determined, 4 (0.76%) had an HLA-B*5701 allele. This is consistent with the finding of a previous study which demonstrated that 2 of 813 (0.25%) Yi nationality carried HLA-B*5701 allele in Liangshan Autonomous Prefecture, Sichuan Province, China.

We also investigated the prevalence of HLA-B*5701 in subjects born from different areas. Our results demonstrated that there was no difference in prevalence of HLA-B*5701 across China. Low prevalence is also found in other area of East Asia. Saito et al. [7] showed that none of 371 Japanese expressed HLA-B*5701, and Park et al. [8] reported that No patients had the HLA-B*5701 allele in 534 HIV infected Korean patients. However, in some area of Southern Asia, the carriage rates of HLA-B*5701 were relatively higher, such as 1.7% in India [17] and 4% in Thailand [18].

The prevalence of suspected ABC hypersensitivity was 9–10% in Caucasians not routinely screened for HLA-B*5701. Prospective screening for HLA-B*5701 prior to initiation of ABC can significantly reduce the incidence of ABC hypersensitivity in Caucasians [11–13]. Such practice appears to be cost-effective in terms of healthcare resources. However, application of routine HLA-B*5701 testing to other racial populations deserves further investigation. In East Asia, the incidence of clinically suspected ABC hypersensitivity was relatively low (4–5%) [6, 8] in HIV infected patients not screened for HLA-B*5701, which was comparable to that in Caucasians (4%) who were prospectively screened for HLA-B*5701 [1]. The low incidence of ABC hypersensitivity might be explained by significantly lower genetic frequency of HLA-B*5701. In addition, ABC hypersensitivity might occur in HLA-B*5701-negative patients [6, 19], the prevalence of clinically suspected ABC hypersensitivity did not decrease even after HLA-B*5701 screening was introduced [8]. Thus, utilization of routine testing for HLA-B*5701 would not be a cost-effective strategy to reduce the risk associated with ABC hypersensitivity in China. However, ABC hypersensitivity is a serious condition, clinical training on early detection of the clinical symptoms of abacavir hypersensitivity, especially in the first 6 weeks of treatment, should be emphasized.

The limitation in our study is that positive and undetermined samples were only confirmed using the same method. It would be better to retest the samples by other method, such as sequence-based typing. However, PCR-SSP is a common used method in HLA typing. According to the instruction, the commercial kit we used is designed to amplify all HLA-B*57 alleles in eight PCR reactions and unequivocally identify B*57:01. Our study may provide important information about the prevalence of HLA-B*5701 in China.

## Conclusion

HLA-B*5701 is very rare in Chinese patients with HIV infection, whether in Han or Non-Han Chinese populations. And there are no significant differences among different areas of China. HLA-B*5701 screening may not be a cost-effective strategy to reduce the risk associated with ABC hypersensitivity in China.

## References

[1] Mallal S, Phillips E, Carosi G, Molina JM, Workman C, Tomazic J (2008). HLA-B*5701 screening for hypersensitivity to abacavir. N Engl J Med.

[2] Hughes AR, Mosteller M, Bansal AT, Davies K, Haneline SA, Lai EH (2004). Association of genetic variations in HLA-B region with hypersensitivity to abacavir in some, but not all, populations. Pharmacogenomics.

[3] Panel on Antiretroviral Guidelines for Adults and Adolescents (2015) Guidelines for the use of antiretroviral agents in HIV-1-infected adults and adolescents. Department of Health and Human Services. Available at http://aidsinfo.nih.gov/contentfiles/lvguidelines/AdultandAdolescentGL.pdf. Accessed 27 June 2015

[4] Clumeck N, Pozniak A, Raffi F (2008). European AIDS Clinical Society (EACS) guidelines for the clinical management and treatment of HIV-infected adults. HIV Med.

[5] Nolan D, Gaudieri S, Mallal S (2003). Pharmacogenetics: a practical role in predicting antiretroviral drug toxicity?. J HIV Ther.

[6] Sun HY, Hung CC, Lin PH, Chang SF, Yang CY, Chang SY (2007). Incidence of abacavir hypersensitivity and its relationship with HLA-B*5701 in HIV-infected patients in Taiwan. J Antimicrob Chemother.

[7] Saito S, Ota S, Yamada E, Inoko H, Ota M (2000). Allele frequencies and haplotypic associations defined by allelic DNA typing at HLA class I and class II loci in the Japanese population. Tissue Antigens.

[8] Park WB, Choe PG, Song KH, Lee S, Jang HC, Jeon JH (2009). Should HLA-B*5701 screening be performed in every ethnic group before starting abacavir?. Clin Infect Dis.

[9] Phillips EJ (2006). Genetic screening to prevent abacavir hypersensitivity reaction: are we there yet?. Clin Infect Dis.

[10] Orkin C, Sadiq ST, Rice L, Jackson F (2010). Prospective epidemiological study of the prevalence of human leukocyte antigen (HLA)-B*5701 in HIV-1-infected UK subjects. HIV Med.

[11] Rauch A, Nolan D, Martin A, McKinnon E, Almeida C, Mallal S (2006). Prospective genetic screening decreases the incidence of abacavir hypersensitivity reactions in the Western Australian HIV cohort study. Clin Infect Dis.

[12] Waters LJ, Mandalia S, Gazzard B, Nelson M (2007). Prospective HLA-B*5701 screening and abacavir hypersensitivity: a single centre experience. AIDS.

[13] Zucman D, Truchis P, Majerholc C, Stegman S, Caillat-Zucman S (2007). Prospective screening for human leukocyte antigen-B*5701 avoids abacavir hypersensitivity reaction in the ethnically mixed French HIV population. J Acquir Immune Defic Syndr.

[14] Li X-M, Yao J, Ma M-J, Wei D-Y, Lv C, Zhang C-G (2011). Prevalence of human leukocyte antigen B site 5701 allele (HLA-B*5701) in HIV infected intravenous drug users (IDUs) in Liangshan of Sichuan province. Chin J AIDS STD.

[15] Middleton D, Hawkins BR, Williams F, Meenagh A, Moscoso J, Zamora J (2004). HLA class I allele distribution of a Hong Kong Chinese population based on high-resolution PCR-SSOP typing. Tissue Antigens.

[16] China NBoSo (2011) 2010 Sixth national population census main data bulletin (no. 1). http://www.stats.gov.cn/tjgb/rkpcgb/qgrkpcgb/t20110428_402722232.htm. 2011

[17] Chaudhari DV, Chavan VR, Ahir SP, Kerkar SC, Mehta PR, Mania-Pramanik J (2013). Human leukocyte antigen B distribution in HIV discordant cohort from India. Immunol Lett.

[18] Puthanakit T, Bunupuradah T, Kosalaraksa P, Vibol U, Hansudewechakul R, Ubolyam S (2013). Prevalence of human leukocyte antigen-B*5701 among HIV-infected children in Thailand and Cambodia: implications for abacavir use. Pediatr Infect Dis J.

[19] Calza L, Rosseti N, Biagetti C, Pocaterra D, Colangeli V, Manfredi R (2009). Abacavir-induced reaction with fever and severe skin rash in a patient tested human leukocyte antigen-B*5701 negative. Int J STD AIDS.

